# Genomic phenotyping of the essential and non-essential yeast genome detects novel pathways for alkylation resistance

**DOI:** 10.1186/1752-0509-5-157

**Published:** 2011-10-06

**Authors:** J Peter Svensson, Laia Quirós Pesudo, Rebecca C Fry, Yeyejide A Adeleye, Paul Carmichael, Leona D Samson

**Affiliations:** 1 Biological Engineering Department, Center for Environmental Health Sciences, Biology Department, Koch Institute for Integrative Cancer Research, Massachusetts Institute of Technology, Cambridge, MA 02139, U.S.A; 2 Safety & Environmental Assurance Centre (SEAC), Unilever, Colworth Science Park, Sharnbrook, Bedfordshire MK44 1LQ, UK; 3 Department of Biosciences and Nutrition, Karolinska Institutet, 14183 Huddinge, Sweden; 4 Department of Environmental Sciences and Engineering, Gillings School of Global Public Health, Chapel Hill, NC 27599, U.S.A

## Abstract

**Background:**

A myriad of new chemicals has been introduced into our environment and exposure to these agents can damage cells and induce cytotoxicity through different mechanisms, including damaging DNA directly. Analysis of global transcriptional and phenotypic responses in the yeast *S*. *cerevisiae *provides means to identify pathways of damage recovery upon toxic exposure.

**Results:**

Here we present a phenotypic screen of *S*. *cerevisiae *in liquid culture in a microtiter format. Detailed growth measurements were analyzed to reveal effects on ~5,500 different haploid strains that have either non-essential genes deleted or essential genes modified to generate unstable transcripts. The pattern of yeast mutants that are growth-inhibited (compared to WT cells) reveals the mechanisms ordinarily used to recover after damage. In addition to identifying previously-described DNA repair and cell cycle checkpoint deficient strains, we also identified new functional groups that profoundly affect MMS sensitivity, including RNA processing and telomere maintenance.

**Conclusions:**

We present here a data-driven method to reveal modes of toxicity of different agents that impair cellular growth. The results from this study complement previous genomic phenotyping studies as we have expanded the data to include essential genes and to provide detailed mutant growth analysis for each individual strain. This eukaryotic testing system could potentially be used to screen compounds for toxicity, to identify mechanisms of toxicity, and to reduce the need for animal testing.

## Introduction

The DNA damage response in budding yeast *S*. *cerevisiae *is well characterized, especially regarding its response to the alkylating agent methyl methanesulfonate (MMS) [1-8]. In addition to the ~150 yeast proteins directly involved in DNA repair [9], a plethora of proteins with other biological functions are necessary for recovery after damage [1,2]. The mechanistic relevance of many of these proteins in cellular recovery is still not fully understood. Yeast, as a eukaryotic model system, serves as an eminent tool to develop new methods to unravel pathways for modulating the toxicity of agents, especially those agents with unknown modes of action. Several tests, such as the Ames test or the RAD54-GFP Greenscreen [10], exist to determine the genotoxicity of compounds. However, these tests do not always reveal the agents' modes of genotoxicity or the consequential cellular responses elicited by the interactions between the agent and cellular components other than DNA. In addition, these tests are notorious for false positives in predicting the toxicity of an agent for mammalian cells, as revealed later by animal testing. To decipher the mode of toxicity by different toxicants, powerful tools such as genomic phenotyping have been developed [1,2,11-16]. Such methodology is used to determine growth under various conditions for an entire panel of 4,852 yeast strains with single non-essential genes deleted. Of the estimated 6,000 genes in *S. cerevisiae*, 80% are non-essential for growth in rich media; the remaining are essential genes that cannot be deleted and are thus more difficult to study. The subset of essential genes is more highly conserved between species [17] and may therefore be of more relevance in understanding how humans react to toxicants. Essential genes can be studied in hemizygous diploid strains [18] and in haploid strains with either conditional expression of genes or with decreased levels of transcripts [19,20]. We have queried the essential genes in the Decreased Abundance by mRNA Perturbation (DAmP) library of haploid strains [19,21]; transcript levels in the DAmP library were reduced by tagging the 3' UTR of the transcripts with a sequence that elicits nonsense-mediated decay [22].

By using arrayed assays of growing liquid cultures in a microtiter format, sensitive detection of toxicity is achieved. Previous studies using liquid assays in microtiter plates were not high throughput enough to allow screening of the entire yeast genome [23], and although high throughput analysis has been achieved by others, that was only by pooling strains tagged with a specific DNA sequence 'bar-code'. That method detects differences in fast-growing strains, but slow-growing strains are depleted from the pool and are thus quantified with less precision. However, this obstacle may be overcome by deep sequencing of the 'bar-codes' instead of the more common detection by microarrays [24,25].

Here we present a sensitive yet robust and highly automated liquid culture method that we have used as a screen to reveal modes of damage recovery in a eukaryotic system. By combining our data with protein-protein interaction maps, and using databases of functional categories, we have discovered novel biological pathways important for the recovery of cells in response to toxicants. Importantly, the screen has the potential to increase our understanding of toxicity modulating pathways for many different agents. The eukaryotic testing system we present here could be used to screen novel compounds for toxicity and thus reduce the need for animal testing.

## Results

### Experimental system to query genotoxic agents

**Figure 1:**
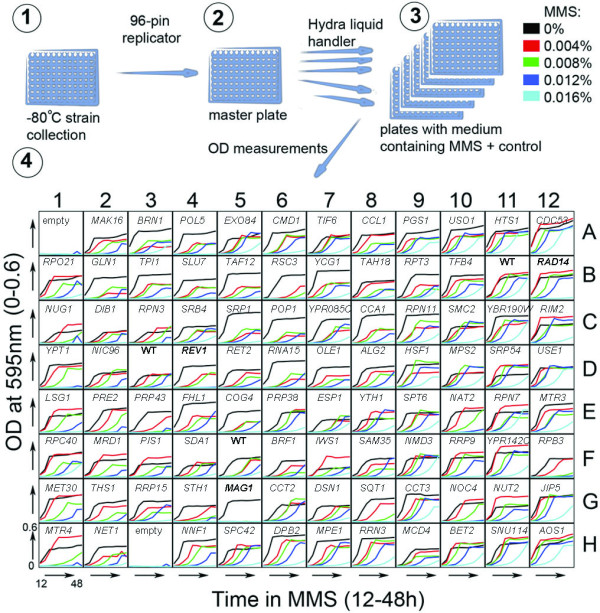
**Schematic of the experimental procedure**. (1) Cells are kept at -80°C from where they are pin-replicated and (2) grown to stationary phase in a master plate. (3) Once in stationary phase, the cultures are robotically diluted in YPD media (using a Hydra liquid handler) containing increasing doses of MMS (0-0.016% final concentration). (4) After incubation at 25°C for 12 hours, optical densities of all cultures are measured every 4 h until 48 h post-treatment. Growth curves are plotted and data is analyzed. As an example, plate 1 from the DAmP library is shown with the name of gene deleted in each strain given above the growth curves. Every plate contains control strains (in bold), WT (B11, D3, F5), *rad14Δ *(B12), *rev1Δ *(D4) and *mag1Δ *(G5), as well as at least two empty wells, containing media only.

**Figure 2:**
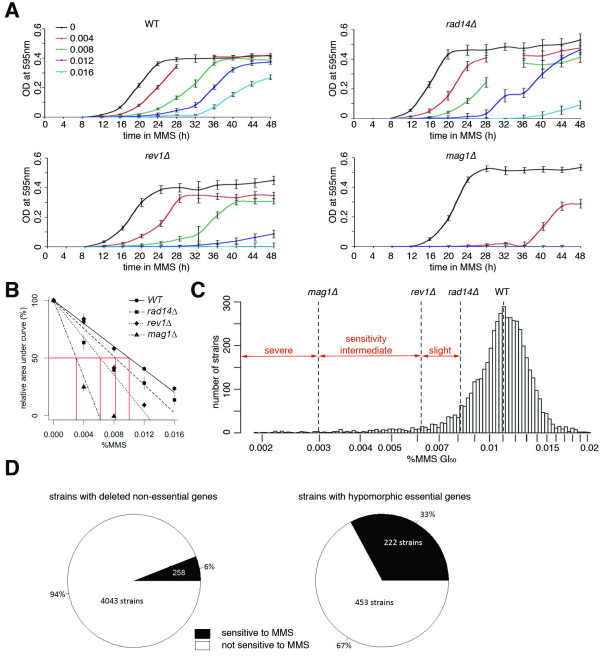
**Determination of sensitivity phenotype of the tested strains**. A) Growth curves of the controls - WT and the sensitive strains *rad14Δ, rev1Δ*, and *mag1Δ *- during 12-48 h after insult with increasing doses of alkylating agent MMS. A few data points at 32 h were undersampled and showed large variation. These points were omitted from the graphs. B) The area under the growth curves were plotted against the dose to calculate the dose that result in 50% growth inhibition (GI_50_). The data for the four control strains are shown. Error bars represents the s.e.m. C) Histograms of showing the distribution of GI_50 _from the entire tested panel (both deletion and DAmP strains), with the GI_50 _of the four control strains forming the limits between severe, intermediate and slight sensitivity to MMS. The WT strain has been transfected with a plasmid conferring G418 resistance that alters the sensitivity of the strain slightly. D) Pie charts of sensitivities of the strains lacking (left) non-essential genes (deletion strains) and (right) essential genes (DAmP strains).

To systematically characterize biological responses to toxic agents, we set up a system where yeast strains were exposed to increasing doses of the alkylating agent MMS. Mutations in 5,528 *S. cerevisiae *genes, representing ~ 92% of the *S. cerevisiae *genome [26], are now available as individual haploid yeast strains; each strain carries either a complete gene deletion or an insertion in the 3' UTR of a gene to destabilize its transcript, thus reducing its steady state level [19,21]. The yeast strains were grown in 96 well plates to stationary phase and then diluted into MMS-containing media (0, 0.004, 0.008, 0.012 and 0.016% MMS). Growth kinetics were recorded in the presence of the DNA damaging agent by optical density measurements at room temperature every 4 hours between 12 and 48 h after transfer to MMS-containing medium (Figure 1), and every strain was queried in at least three independent experiments. Three control strains with known MMS sensitivity were present in every plate: *rad14Δ*, *rev1Δ *and *mag1Δ *were present at three known locations, and WT was present at three other locations (Figure 1, 2A). The dose response for each strain was calculated by determination of the area under the growth curve at each dose (see Methods section for details). In this study, the dose response was required to behave in a close-to linear manner to be considered valid, such that only when the dose response of a strain could be fitted to a straight line (R^2^>0.7) were the data included (Figure 2B). For 89.3% of the deletion strains, and 76.9% of the DAmP strains, two or more experiments fulfilled this criterion, resulting in 87.3% coverage of the tested 5,528 yeast strains.

For each strain, the dose that gave 50% growth inhibition (GI_50_) was calculated based on a total of a median of 150 measurements (10 time points × 5 doses × 2-4 replicates). Only replicates that passed the quality criterion above of R^2^>0.7 were included in the calculations. The GI_50 _for WT haploid yeast was determined as 0.01 ± 0.002% MMS (average ± s.e.m.). The GI_50 _for the three control strains were 0.008 ± 0.001%, 0.006 ± 0.0005%, and 0.003 ± 0.0007% for *rad14Δ*, *rev1Δ*, and *mag1Δ *respectively (Figure 2B). The reproducibility between experiments was high (the average R^2 ^for the GI_50 _between replicates was 0.88 ± 0.03). To make a quantitative comparison of the tested strains to previous studies, all strains were categorized as showing severe, intermediate, and slight or no sensitivity to MMS. This categorization was based on the comparison between GI_50 _values of the tested strains, WT and the three control strains used as standards to indicate the thresholds for slight (*rad14Δ*), intermediate (*rev1Δ*) and severe (*mag1Δ*) sensitivity (Figure 2C).

In total, 258 (6%) of the 4,331 deletion strains that passed the quality criterion were determined to be more sensitive to MMS than WT; among these strains 18 (7.0%) showed severe, 87 (33.7%) showed intermediate, and 153 (59.3%) showed slight MMS sensitivity (Figure 2D, Table S1, Additional file 1). A much higher fraction of the DAmP strains with hypomorphic mutations in essential genes demonstrated sensitivity to MMS compared to the deletion strains. Among the 675 DAmP strains that passed the quality criterion, 222 (33%) were MMS sensitive; among these strains 13 (5.9%) showed severe, 69 (31.1%) showed intermediate, and 140 (63.1%) showed slight MMS sensitivity (Figure 2D, Table S1, Additional file 1). The environmental stress response (ESR) genes [27] only modestly overlap with the genes deleted in the MMS sensitive strains; the ESR genes make up 19% of the genes deleted in sensitive strains, whereas the ESR comprises 16% of the entire genome (p = 0.002). Further, the WT used here was a modified version of BY4741 (with a plasmid conferring G418 resistance). This strain was confirmed as being slightly more sensitive than the original BY4741 (p-value < 0.01, t-test). The assay has its most sensitive range in detecting strains with GI_50 _between 0.002 and 0.008, where the data points to calculate the GI_50 _cover the entire range from control growth (100%) to no growth (0%). The confidence in calculating the GI_50 _of resistant strains decreases, as the growth is not as inhibited by the tested doses. However, we also identified 152 strains from both libraries (145 deletion strains, 7 DAmP strains) that showed some resistance to MMS compared to WT (Figure S1, Additional file 2). The criterion for resistance is described in the Methods section. No GO term was significantly enriched (FDR<0.05) among the genes that conferred resistance when deleted. As in previous studies, this method has been unable to reproducibly identify resistant strains [1,2].

The data was also used to calculate the time required for the cultures to demonstrate visible growth (lag time) and the MMS dependency of the lag time (Figure S2, Additional file 2). Most strains, including the four control strains had a lag time 10-0 h. "Slow-growers" were defined as having a lag time exceeding 20 h. A large fraction (40%) of the "slow-growers" came from the relatively small DAmP library. Among the sensitive strains, a significant proportion (26.0%) were "slow-growers", which is significantly higher (p < 10^-47^) than in the entire collection (6.7%). This observation was further confirmed by a comparison with other "slow-growers" identified elsewhere [28]. In this set 18.6% of the sensitive strains were identified as "slow-growers", again significantly higher (p < 10^-9^) than in the entire collection (8.6%). The MMS dependency of the lag time represents an alternative measure of MMS sensitivity (Figure S2C, Additional file 2).

The MMS sensitivities and the lag times of the individual strains in this liquid assay are listed in Table S1 and S2 (Additional file 1).

### Functional enrichment

To determine the biological functions involved in the recovery after being exposed to the DNA damaging agents MMS, we sought enrichment among gene ontology (GO) functional categories (see Methods) in the selection of genes deleted in the MMS sensitive strains compared to the entire genome. Among the functions that are highly enriched in the MMS sensitive strains are, as expected, DNA repair, cell cycle and transcription, but in addition the unexpected categories of telomere maintenance and RNA processing are also very highly enriched in the MMS sensitive strains (abstracted data in Table 1 complete data in Table S3, Additional file 1). Previous genomic phenotyping on solid agar did not find 'telomere maintenance' enriched among MMS sensitive strains [2]. However, re-analysis of the previous data with updated gene annotations revealed that this function and related GO categories are in fact among the most significantly enriched terms in that data set (Table S4, Additional file 1). Moreover, the RNA processing functions revealed in the current study derive primarily from screening the essential genes, which was not possible at the time of the previous study [2].

**Table 1:** Enriched GO terms among the toxicity-modulating genes

GO IDDescription		P-value	Adjusted P-value	x/X* (%)	n/N** (%)
6974	response to DNA damage stimulus	6.8E-19	2.1E-16	15.3	4.8
6259	DNA metabolic process	3.0E-16	4.7E-14	19.0	7.6
6281	DNA repair	2.9E-16	4.7E-14	12.8	3.9
22402	cell cycle process	6.6E-12	5.4E-10	17.4	7.9
6394	RNA processing	2.5E-09	1.5E-07	16.0	7.9
7001	chromosome organization and biogenesis	4.4E-09	2.4E-07	14.4	6.8
6365	rRNA processing	1.3E-08	6.4E-07	9.2	3.5
6260	DNA replication	1.4E-08	6.5E-07	7.8	2.7
278	mitotic cell cycle	2.2E-08	9.3E-07	11.4	5.1
6351	transcription, DNA-dependent	2.4E-08	9.8E-07	11.2	4.9
6302	double-strand break repair	2.5E-08	9.8E-07	4.3	1.0
34470	ncRNA processing	3.4E-08	1.3E-06	11.2	5.0
65004	protein-DNA complex assembly	1.1E-07	3.7E-06	5.9	1.9
723	telomere maintenance	2.0E-05	3.7E-04	3.4	1.0
6395	RNA splicing	2.4E-05	4.4E-04	5.7	2.4
7059	chromosome segregation	4.0E-04	5.0E-03	5.3	2.5
51656	establishment of organelle localization	3.9E-03	3.4E-02	2.5	1.0
7127	meiosis I	4.4E-03	3.8E-02	3.0	1.3

* number of toxicity-modulating genes with GO term/number of toxicity-modulating genes × 100

** number of genes with GO term/number of genes × 100

Table only shows non-redundant GO terms containing between 50 and 500 genes, with FDR adjusted p-value <0.05 and an enrichment of at least 2. For complete table, see supplemental table S3 (Additional file 1).

### Protein-protein interaction networks

**Figure 3:**
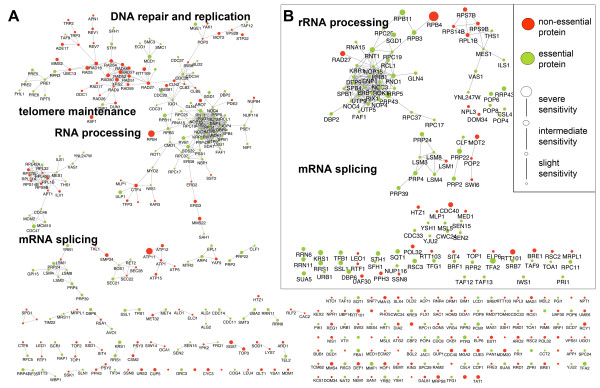
**Yeast protein-protein interaction networks**. A) Network of proteins whose absence or reduced levels render cells sensitive to MMS B) Sub-network of these proteins involved in RNA processing. Non-essential proteins are in red, essential proteins in green. The size (inverse to the GI_50_-value) reflects the sensitivity to MMS of the corresponding yeast strains. Classification of severe, intermediate and slight sensitivity was established according to GI_50 _values (GI_50_<0.003%, GI_50_<0.006% and GI_50_<0.008%, respectively).

To interpret the results in a wider biological context, the protein products of all toxicity-modulating genes were mapped onto the *S. cerevisiae *protein-protein interaction network [29]. One large interconnected network with several well-defined sub-networks were identified (Figure 3A). As previously assumed [2], non-essential and essential toxicity-modulating gene products were present in the same biological networks. The networks were highly connected as each protein had an average number of 2.2 protein-protein interactions (p < 0.001, permutation test). In a random sample of equal size, each protein had on average 1.2 protein-protein interactions. The identified sub-networks are discussed below.

#### DNA repair and replication

As expected, many strains deficient in DNA repair proteins are identified as MMS sensitive. In concordance with previous results [1,2], this set of toxicity-modulating genes include members of the *RAD52 *epistasis group, such as *RAD50, RAD51, RAD52, RAD59, RAD54 *and *RAD57*. Together with *XRS2*, these gene products are required for homologous recombination repair of DNA double-strand breaks (reviewed in [30]). Other DNA repair pathways that were important for cellular recovery after MMS include both the base excision repair pathway (*MAG1, APN1*) and the nucleotide excision repair pathway (*RAD14, SSL1, TFB1, RAD26*), including associated factors (*RAD9, RAD24, DEF1*). Rad14p is the yeast homolog of damage binding protein XPA [31], and Ssl1p and Tfb1p are subunits of the TFIIH complex essential for NER [32,33]. Rad9p and Rad24p are checkpoint proteins required for NER [34]. A branch of NER, transcription coupled repair (TCR), is effective on the transcribed strand of DNA. This pathway is represented by *RAD26 *and *DEF1*. Rad26p is the yeast homolog of CSB, a DNA dependent ATPase [35] and Def1p is required for the ubiquitination and subsequent proteolytic degradation of RNA pol II [36,37]. The involvement of transcription coupled repair for DNA methylation damage is surprising in light of previous reports stating that TCR does not act on methylated DNA bases in mammalian cells [38]. The post-replication repair error-free prone pathway was also represented in our dataset, albeit by mutants that showed only slight MMS sensitivity (*POL32, MMS2, RAD6, UBC13, RAD52, RAD5, RAD18*).

We also identified three essential genes that encode three subunits of the Replication Factor C (RFC), namely *RFC5*, *RFC1 *and *RFC3*, as being important for MMS-induced damage recovery. The RFC complex is involved in both DNA repair and DNA replication, acting as a "clamp loader" to load Pol30p (the yeast homolog of PCNA) onto DNA; RFC is also thought to contribute to the maintenance of a DNA replication checkpoint during S phase [39].

#### Telomere maintenance

DNA damaging agents can induce gross chromosomal rearrangements and MMS is known to induce chromosomal aberrations in the form of telomere additions and translocations [40]. In the last few years, various genome-wide screens have shown that more than 350 genes affect the regulation of telomere length [41-43]. The *RAD52 *epistasis group provides a telomerase-independent mechanism of telomere maintenance, and is heavily represented among the toxicity-modulating proteins, as mentioned above. Besides the *RAD52 *epistasis group, deletion of other non-essential genes involved in telomere maintenance also results in MMS sensitivity. For instance, severe MMS sensitivity results upon deletion of *SGS1 *encoding a DNA helicase of the RecQ family that is required for recombination-mediated telomere lengthening [44,45]. The Sgs1p N-terminal physically interacts with Top3p [46] and Rmi1p [47], two other proteins that when lacking cause severe cellular sensitivity to MMS. Intermediate sensitivity results from deletion of *EST1*, encoding a protein associated with the telomere template RNA sequence (*TLC1 *RNA) used to add TG-repeats to form telomeric DNA that is part of the telomerase complex, and is essential for effective telomerase function [43,48]. Deletion of *YKU80 *also results in intermediate MMS sensitivity; this gene encodes a subunit of the Ku heterodimer, a DNA repair complex that also binds *TLC1 *RNA [40].

DAmP mutations in three essential genes related with 'telomere maintenance' were also found to result in a MMS sensitive phenotype. These essential genes were as follows: *RAP1*, encoding a protein that caps chromosome ends to prevent telomere fusion [49,50]; *TEL2*, encoding a protein that binds specifically to single-stranded telomeric DNA repeats and is required for telomere length regulation and telomere position effect [51]; *DDC2*, encoding a protein that interacts directly with Mec1p and Mec3p that are part of the essential component of the telomere checkpoint pathway, activated in the presence of DNA damage to induce a delay in cell cycle progression [52].

#### RNA processing

One of the major categories of cellular functions for essential genes is RNA processing. Approximately 10% of the entire *S. cerevisiae *genome is involved in one of various RNA-related processes [53], including mRNA splicing and export, tRNA modification, translation, rRNA processing, and RNA degradation. By screening the essential genes in the DAmP library, we found the GO term 'RNA processing' highly enriched among the toxicity-modulating proteins. A total of 61 strains sensitive to MMS had defects in proteins associated with this biological process; these are integrated within 2 subnetworks (Figure 3B). One network comprised of 20 essential proteins is primarily involved in rRNA processing and ribosome biogenesis (Figure 3B). Among this set there are two proteins that convey severe sensitivity to MMS when levels are reduced, namely Rnt1p and Prp43p. Rnt1p is an RNA endonuclease and Prp43p is an RNA helicase; both are involved in cleavage of the 3'-end of pre-rRNAs [54-56]. Prp43p is also involved in the release of lariat-introns from the spliceosome processing of pre-mRNAs [57]. As described below, many more proteins involved in mRNA splicing were shown to affect the recovery of cells from MMS-induced damage.

#### mRNA splicing

Seventeen proteins in the sub-networks are involved in nuclear mRNA splicing via the spliceosome (Figure 3B). mRNA splicing is a complex reaction involving dozens of proteins, and consisting of two consecutive catalytic reactions divided into three coordinated stages [58]. Toxicity-modulating genes were found to be involved in each of the three stages as follows: in the assembly and activation of the spliceosome (*CDC40, BRR2, CLF1, LSM4, LSM8, PRP40, SMX3, PRP39*); in the catalysis stage (*PRP4, MSL5, PRP2, PRP24, DIB*, *SNU56, YHC1*); and in the release, disassembly and snRNP recycling stage (*PRP43 *and *PRP22*).

In addition to mRNA splicing, MMS sensitivity was produced upon reduced expression of genes involved in other kinds of RNA splicing, such as snoRNA splicing (*CWC24*) and tRNA splicing (*PTA1, NUP116, NUP49, SEN15, SEN2, POP1, POP6, POP4, RPR2, SPB1*). The surprising finding that RNA splicing of all kinds is required for cellular survival after damage with MMS could be a reflection of the need for spliced gene products to help cells recover. In *S. cerevisiae *only ~ 280 genes contain introns (5% of all genes). Twenty of the 506 toxicity-modulating genes have introns (table 2), corresponding to ~ 4%, indicating no significant enrichment for intron-containing genes among the toxicity-modulating genes. While most intron-containing genes are implicated in the ribosomal machinery, and some are involved in meiosis [59], there was no significant enrichment of any biological process among the 20 toxicity-modulating intron-containing genes. However, it should be noted that three of these 20 gene products have well-described roles in DNA repair (Mms2p, Ubc13p and Rad14p), suggesting a highly specific role of these particular spliced gene products after DNA damage. Yeast strains with any one of these three genes deleted are sensitive to a number of genotoxic agents, including MMS, 4-NQO and UV [2]. Exactly why so many different kinds of RNA processing are important for the recovery of cell growth after exposure to MMS is not yet clear, but the extent to which the RNA-processing deficient mutants are sensitive and the fact that so many kinds of RNA are involved points to a fundamentally important biological mechanism. We are currently investigating why deficient RNA processing of various types renders cells so sensitive to DNA damaging agents.

**Table 2:** Toxicity-modulating genes containing introns.

ORF	Common name	GI_50 _(%MMS)
YGL087C*	MMS2	0.004
YDR367W		0.005
YDR092W*	UBC13	0.005
YNL096C**	RPS7B	0.005
YNL038W	GPI15	0.006
YNL112W	DBP2	0.006
YLL050C	COF1	0.006
YDL075W*	RPL31A	0.007
YFR045W*		0.007
YBL018C	POP8	0.007
YJL191W**	RPS14B	0.007
YMR033W	ARP9	0.008
YKR094C**	RPL40B	0.008
YMR079W	SEC14	0.008
YMR116C*	ASC1	0.008
YNL162W**	RPL42A	0.008
YLR078C	BOS1	0.008
YHR041C*	SRB2	0.008
YML094W*	GIM5	0.008
YMR201C*	RAD14	0.008

* Sensitive in Begley et al 2004.

** Not sensitive in Begley et al 2004.

### Reproducing previous data

**Figure 4:**
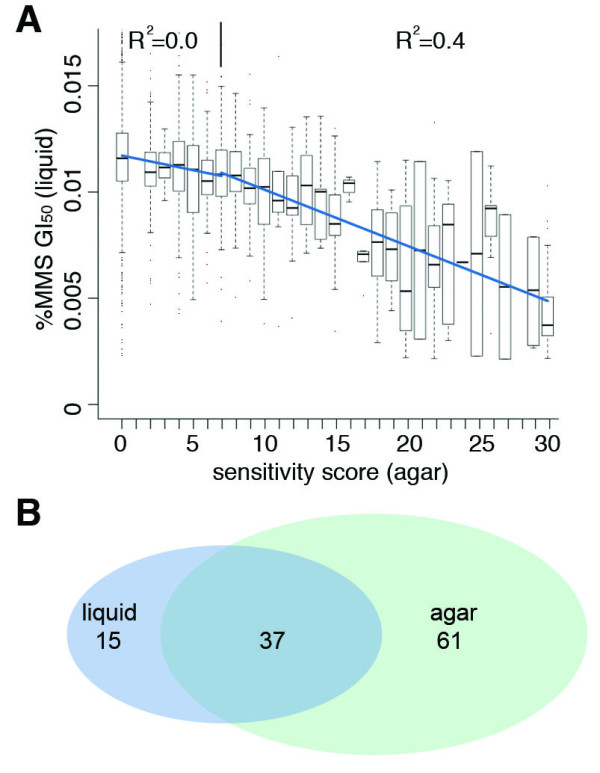
**Comparison between results of liquid genomic phenotyping (this study) and previous results using a solid agar assay (Begley et al, 2004)**. A) A boxplot of the previous dataset (x-axis) where the data ranges from 0 (no sensitivity to MMS) and 30 (high sensitivity to MMS) and the dataset presented here. The bold line represents the median, the box contains 50% of the data, the whiskers extend to 1.5 times the inter-quartile range, and outliers are represented by dots. Two lines (blue) are fitted with linear regression to the data, one in the range of sensitivity scores 0-7 and one in the range of sensitivity scores 7-20. B) Venn diagram showing the overlap in GO terms enriched in sensitive strains from the liquid assay and the agar assay.

The results obtained in this liquid culture-based screen were compared to previous results using an agar-based colony growth screen [2]; we found reasonable concordance between the datasets for the sensitive strains, especially for the strains with a sensitivity score higher than 7, the score of *rad14Δ *(R^2 ^= 0.4). The strains with a sensitivity score less than 7 are not reliably detected in the liquid assay (line fitted to the data with R^2 ^= 0.0) (Figure 4A). In the previous data set, only the non-essential genes were tested by growing individual yeast strains on solid agar; each strain was assigned a sensitivity score ranging from 0 (no sensitivity) to 30 (highest sensitivity) based on the extent of growth on MMS-containing agar relative to the WT strain. Compared to the liquid culture screen, the solid agar screen, which was far more labor-intensive, found many more strains sensitive to MMS. In the current study we defined the thresholds of severe, intermediate and slight sensitivity based on the scores of the three control strains *mag1Δ, rev1Δ *and *rad14Δ*, respectively. After the application of these thresholds, the previous study identified a total 588 out of 4852 deletion strains tested (12%) as being sensitive to MMS: 30 strains showed severe sensitivity, 43 strains showed intermediate sensitivity, and 515 strains showed slight sensitivity. The highest correspondence between the assays is found for strains that show severe or intermediate sensitivity in at least one of the assays. Of the 105 strains with severe/intermediate sensitivity in the liquid assay, 73% (77/105) were also associated with severe/intermediate sensitivity in the agar assay. Among the 73 strains with severe/intermediate sensitivity in the agar assay, 51% (37/73) were determined to have severe/intermediate sensitivity in the liquid assay. Strains with slight MMS sensitivity in the agar screen were within the variation of the WT in the liquid assay presented here. Among the 153 slightly sensitive strains in the liquid assay, 97 (63%) were sensitive in the agar assay, whereof 61 strains showed intermediate sensitivity and 10 strains showed severe sensitivity. Despite the fact that the liquid assay identified fewer sensitive strains than the agar assay (among the non-essential deletion strains) we found 84 strains that had not shown MMS-sensitivity in the agar assay. Among these genes, four were classified as resulting in severe sensitivity when deleted; these are as follows: *TAT1 *(*YBR069C*), an amino acid transporter; *EMP24 *(*YGL200C*), involved in ER to Golgi transport; *YOR331C *encoding a protein of unknown function, localizing to endosomes [60]; and also the as yet uncharacterized *YNL086W*.

Finally, it is very important to note that despite fewer strains being identified in the liquid assay, most (71%) of the enriched functional categories (Bonferroni adjusted p-value<0.0001) in the list of toxicity-modulating genes resulting from the liquid assay (Table S3, Additional file 1) were also found in the list resulting from the reanalysis of previous dataset (Table S4, Additional file 1) (Figure 4B). The main categories uniquely present in the liquid assay can be summarized as processing of different species of RNA, whereas the liquid assay results are lacking a significant enrichment for vesicle transport genes. It should also be noted that since the mutant libraries were screened under different growth conditions (liquid versus agar) we expected to see differences in the pathways detected.

### Different modes of toxicity found through growth patterns

**Figure 5:**
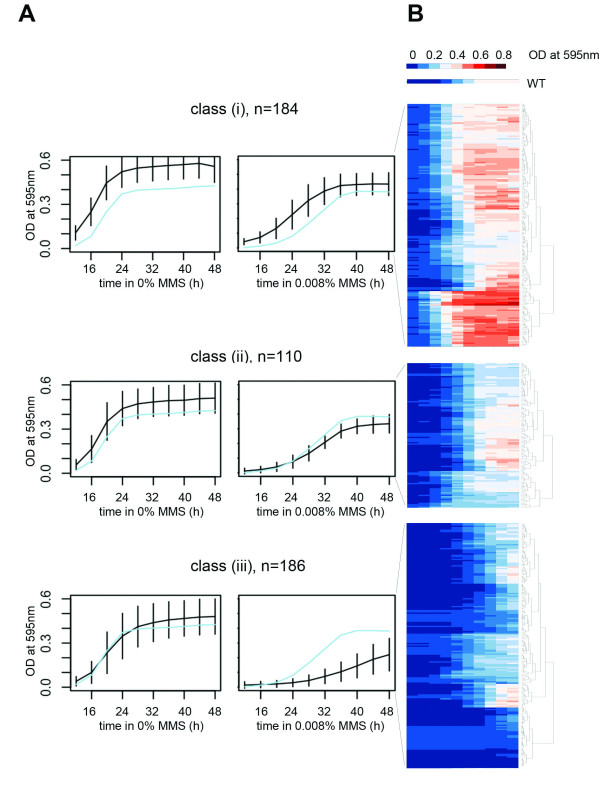
**The strains with intermediate sensitivity are divided into classes based on their growth phenotype after exposure to 0.008% MMS**. A) Through self-organizing maps, three classes were identified based on the growth curves after MMS exposure. WT is shown as a reference in light blue. The black lines show the average values within the class for each time point. B) Hierarchical clustering and heatmaps of the three clusters in A. The growth of the WT strain is shown at the top.

The detailed growth curves obtained in this study allow further categorization of the sensitive yeast strains. The sensitivity phenotype was associated with several distinct growth patterns. The shape of growth curves of each strain in various MMS doses provides a wealth of information (Figure 5). After 0.008% MMS exposure, the WT strain shows a prolonged lag phase, but then starts growing exponentially and reaches the plateau at the same level as the untreated cells (Figure 2A, 5). The growth data of the sensitive strains after 0.008% MMS exposure was subjected to self-organizing map (SOM) analysis to split the data in three classes (Figure 5A). Other doses were also examined but at higher doses, the dynamic range is lost, as many strains do not grow at all; at the lower dose (0.004% MMS), less effect is seen, but the strains still group into similar categories. The three classes evident at the 0.008% MMS dose show distinct growth patterns characterized by: (i) lacking a MMS-induced lag-phase at this dose (n = 184); (ii) showing slower growth compared to WT (n = 110); and (iii) showing a very prolonged lag-phase and a slower recovery rate (n = 186). The average GI_50 _value calculated based on all five doses (0-0.016% MMS) were calculated to be 0.0067 ± 0.0016, 0.0071 ± 0.0010 and 0.0053 ± 0.0016% MMS for clusters (i), (ii) and (iii) respectively (Tables S5-7, Additional file 1). In other words, all three categories are in fact MMS sensitive compared to WT despite the fact that at the 0.008% MMS dose only category (iii) appears to be sensitive, and category (i) appears to be resistant. This underscores the importance of monitoring the effects of a range of doses, and a range of exposure times.

Notably, most of the genes (75/110, 68%) mutated in the class (ii) strains are essential. GO enrichment analysis of the different classes reveals that several functional categories are enriched (Table S8-10, Additional file 1). In particular, class (i) is overrepresented by response to DNA stimulus, DNA repair and DNA replication. The most prominent groups of enrichment in class (ii) are RNA processing and cell cycle. Class (iii) has the most widely distributed functional diversity. The cluster is enriched (FDR<0.05) for 125 GO categories, representing most enriched categories found in the entire dataset of sensitive strains.

The strains in class (iii) are registered by most assays and these results correlate well with previous datasets (78% recognized in our previous study, 51/65 non-essential gene deletion strains). The strains represented in both class (i) and (ii) are expected to be more difficult to detect in assays employing just one late time point to measure sensitivity. However, although class (i) and (ii) show a smaller overlap with previous data than does class (iii), 63% of the non-essential genes in class (i) and 66% of the non-essential genes in class (ii) were in fact detected in the previous dataset that used one late time-point to assess toxicity [2]. Thus, methods relying on a single time point have a slightly lower resolution in detecting the growth patterns of class (i) and (ii). The dynamics of the growth curves make these clusters easy to identify using the method described here.

The complete dataset is available as a database with a web-interface available at http://genomicphenotyping.mit.edu/svensson/2011 (Figure S3, Additional file 2).

## Discussion

In this study, we have measured growth curves after exposure to the DNA damaging agent MMS for a collection of yeast mutant strains deficient in 5,528 essential and non-essential genes. Compared to previous studies using similar genomic phenotyping [1,2], we have expanded the data to include essential genes, and to include detailed growth analysis of each strain; growth was measured at 10 time-points after treatment with a toxicant, in biological triplicates. By testing compounds in a eukaryotic system, an estimate of the toxicity in eukaryotic cells is given, as well as details regarding the way the cell responds to the toxicant, in this case MMS. We show here that genomic phenotyping is a valuable tool to decipher the modes of toxicity conferred by a DNA damaging agent. This was demonstrated by our identification of several novel toxicity-modulating genes, including those involved in RNA processing and telomere maintenance.

The fact that the toxicity-modulating proteins are found within protein-protein interaction networks of significantly higher connectivity than expected (p > 0.001) raises our confidence that the novel candidates are truly needed for cells to recover after MMS-induced damage. This includes the proteins involved in different kinds of RNA processing. It was recently shown that certain tRNA-modifications can influence cell survival after exposure to DNA damaging agents, in both yeast and human cells [61,62]. Here we also identify mRNA, snoRNA and tRNA splicing as being required for survival after DNA damage, even though relatively few yeast transcripts are spliced [59,63]. From this study, it remains inconclusive whether RNA splicing in general is important for helping the cell better handle MMS-induced damage or whether the processing of a few specialized transcripts may provide MMS resistance; such specialized targets include mRNA transcripts from the *MMS2, UBC13 *and *RAD14 *genes, three DNA repair genes all of which are spliced in yeast [59]. However this does not explain why snoRNA and tRNA splicing is required for MMS-resistance.

In addition to genes encoding mRNA, snoRNA and tRNA processing proteins, one of the prominent groups of genes resulting in MMS sensitive strains when deleted, is involved in the rRNA metabolic process, consisting of 'rRNA catabolic process' and 'rRNA processing'. Forty-one out of the 262 (16%) genes of this GO category are toxicity-modulating. The majority of the toxicity-modulating rRNA-related genes are essential in yeast (34/41), which is presumably the reason why these pathways were not identified in previous screens.

Another cellular function highlighted in this study is telomere maintenance. In yeast, many of the telomere maintenance proteins also have functions in DNA damage responses, such as Tel1p and Mec1p, which are homologs of the human ATM and ATR kinases that are activated in response to DNA damage. Yeast telomeres are maintained differently than their metozoan counterparts. The components of the mammalian shelterin complex that protects the telomere ends have no direct homologs in budding yeast, although yeast shelterin-like proteins have been described [64].

The fact that a substantial proportion of the MMS sensitive strains have a slow growing phenotype under normal conditions, could reflect that this subset of the "sensitive" strains are identified as a consequence of the accumulated stress exceeding a viability threshold with the additional DNA damage. However, for the majority of the sensitive strains, this is not the case.

We have further shown that the DAmP strains are very well suited to studying essential genes in this type of damage-sensitivity screening. Given the essential role of these genes, it is not surprising that reduced levels of the transcripts lead to a reduction in growth rate for several of the DAmP strains. Compared to the diploid hemizygous strains [65], the DAmP strains show a higher proportion of toxicity-modulating genes (data not shown). This observation is consistent with previous results using the drug methotrexate [20]. Compared to previous studies of genomic phenotyping, the information provided by this study is richer in data sampling, thus resulting in the possibility to further dissect the modes of toxicity and differentiate between patterns of sensitivity. New modes of sensitivity can be detected through understanding of the dynamics of the growth. Types of sensitivities that could go undetected in other systems can be scored here, as demonstrated by our self-organizing map analysis. Interestingly, the majority of the genes (68%) that were present in class (ii), were essential and primarily members of the relatively small DAmP library. This pattern of MMS sensitivity that is only apparent at higher MMS doses may be explained by the fact that lower levels of transcript expressed in the DAmP may be able to maintain sufficient protein levels to handle low levels of cellular damage but then fail at higher levels of damage.

## Conclusions

To conclude, we present here a data-driven method to reveal modes of toxicity of different agents that impair cellular growth. This eukaryotic testing system could potentially be used to screen compounds for toxicity, to identify mechanisms of toxicity, and to reduce the need for animal testing.

## Methods

### Strains

*S. cerevisiae *strain haploid BY4741, diploid BY4743 were purchased from Research Genetics. As previously described [1], strain BY4741 was transformed with plasmind pYE13g (American Type Culture Collection) which confers G418 resistance. Deletion, DAmP and hemizygous library were purchased from Open Biosystems. The deletion library consists of a collection of 4,852 haploid strains where each strain has a single ORF replaced with the KanMX4 module, which confers G-418 resistance. These strains are in the BY4741 background (*MATa his3Δ leu2Δ met15Δ ura3Δ *).

### Cell culture

96-well master plates containing individual deletion strains were grown to stationary phase in 150ul YPD (10 g yeast extract, 20 g peptone, 20 g dextrose/liter), containing G-418 (Sigma) at 200ug/ml. Three wells of WT yeast and three control strains with known sensitivity were added into the plates. Settled cells were resuspended and a 1600X dilution of the cell suspension was inoculated with five doses (0, 0.004, 0.008, 0.012 and 0.016%) of MMS (Sigma) using a 96-pin Hydra (Robins Scientific). Cells were incubated for 48 h at 30°C. After 12 h, the OD_600 _was measured every 4 h using a Victor3 (Perkin Elmer). Comparison to cultures grown in bulk revealed small differences in growth patterns (data not shown).

### Data analysis

Files with raw data were analyzed with in-house developed scripts in R (http://www.r-project.org). The OD measurements of empty wells were subtracted from all wells. Growth curves for the 48 hours after addition of MMS were drawn for the 5 doses for the individual yeast strains. The area under the curve (AUC) was calculated for each dose (including the mock-treated sample). For each strain, the dose-specific AUC was plotted against the dose. A line was fitted by linear regression and the goodness-of-fit (R^2^) was used to estimate linearity of the response. The slope revealed by the regression was used to determine the dose leading to 50% growth inhibition, GI_50_, by GI_50_= -0.5/slope (Figure 1B). R-scripts to regenerate the analysis are available in supplementary material together with instructions to access the raw data (Additional file 3). The visualization of the heat maps was done in R. Self-organizing maps were implemented through the SOM package. Functional enrichment was performed in Bingo 2.0.

Sensitivity thresholds were calculated based on the average GI_50 _of the three control strains (*mag1Δ, rev1Δ, rad14Δ *). The resistance threshold was determined as GI_50___average _+ (GI_50___average _- GI_50___rad14*Δ*_).

The data is available at a searchable database http://genomicphenotyping.mit.edu/svensson/2011 (Figure S3, Additional file 2).

### Reducing the number of time-points

To assess how essential it was to measure cell density every 4 hours between hour 12 and 48 of the 48 h time course, we determined the loss of information resulting from removal of the data for several time-points (Figure S4, Additional file 2). For practical reasons it is important to note that removal of several measurements at intermediate times had only a limited effect on the reproducibility of the data. The goodness-of fit was 0.97 between the full dataset (with 10 time-points) and a reduced dataset (with six time-points). The coverage was determined by the percentage of tested strains that passed the linearity criterion as R^2^>0.7 using the selected time-points only. Using the more practical six point time-course, the coverage was still 84% versus 89.3% with the full non-essential dataset. On the other hand, only considering single observations (at 24 or 48 h) had drastic negative effects on the reproducibility of the data.

### Network analysis

Yeast interaction networks were retrieved from [29] and loaded into Cytoscape v2.6.1 [66]. Functional enrichment was determined by the plug-in Bingo2.0 [67].

## Competing interests

The authors declare that they have no competing interests.

## Authors' contributions

LDS, RCF and JPS designed the experiments, LQP, JPS conducted the experiments, LQP, JPS, RCF, YAA and PC analyzed the data, LQP, JPS and LDS wrote the manuscript. All authors read and approved the final manuscript.

## Supplementary Material

Additional file 1 **Supplementary tables**. This file includes 10 additional tables to supplement the text. Click here for file

Additional file 2 **Supplementary tables. **This file includes additional figures to supplement the text. Click here for file

Additional file 3 **R script**. Text file containing the R code to regenerate the analysis. Click here for file
