# Supplementary material for: Genomic phenotyping of the essential and non-essential yeast genome detects novel pathways for alkylation resistance
Source: BMC Syst Biol. 2011 Oct 6;5:157. doi: 10.1186/1752-0509-5-157 (PMC3213080; doi:10.1186/1752-0509-5-157)
Supplement: Additional file 2 — Supplementary tables. This file includes additional figures to supplement the text. [file 1752-0509-5-157-S2.PDF]

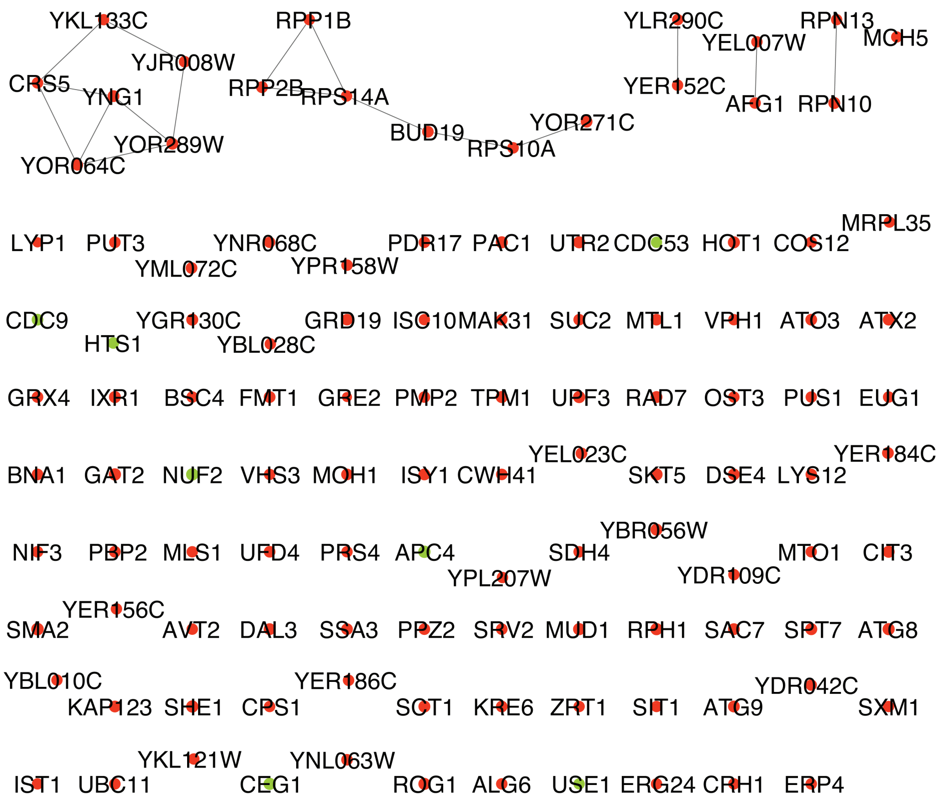

Figure S1. Yeast protein-protein interaction networks of proteins whose absence render cells resistant to MMS. Non-essential proteins are in red, essential proteins in green.

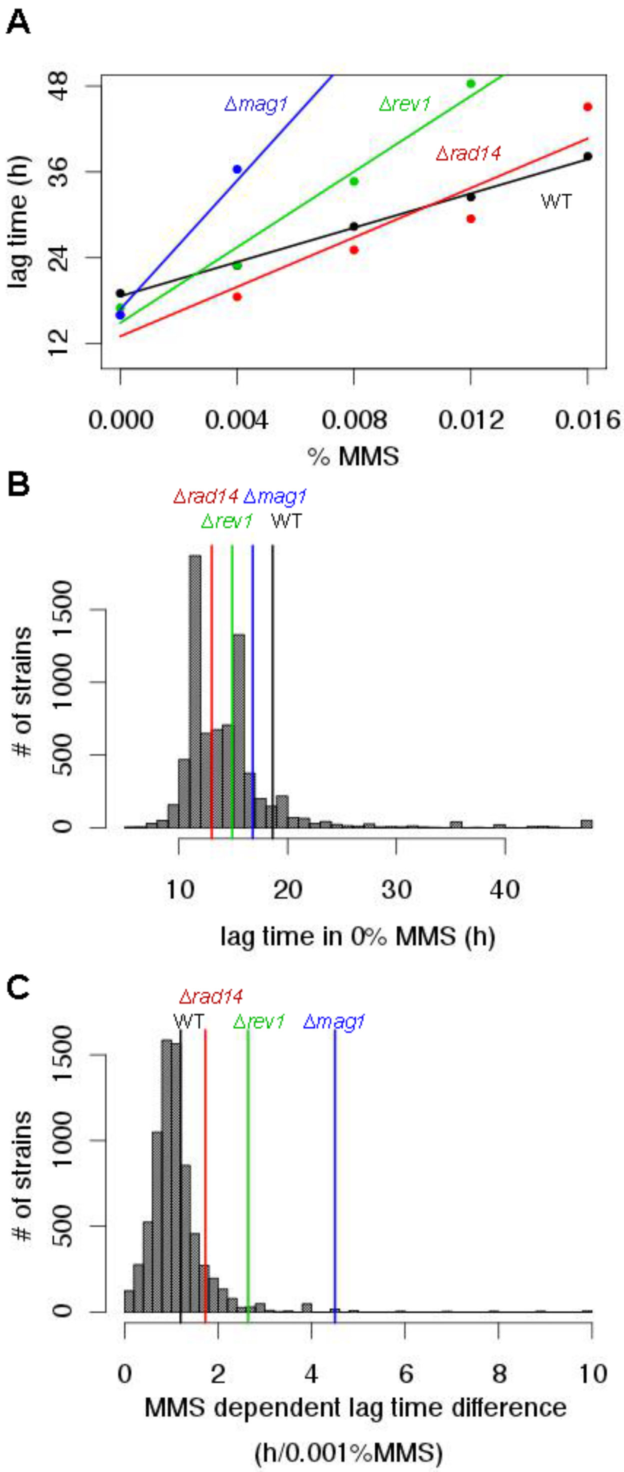

Figure S2. A) Lag time data for the four control strains at the different doses **B)** Histogram of lag times for the entire strain collection **C)** Histogram of the MMS dependency of the lag times for the entire strain collection.

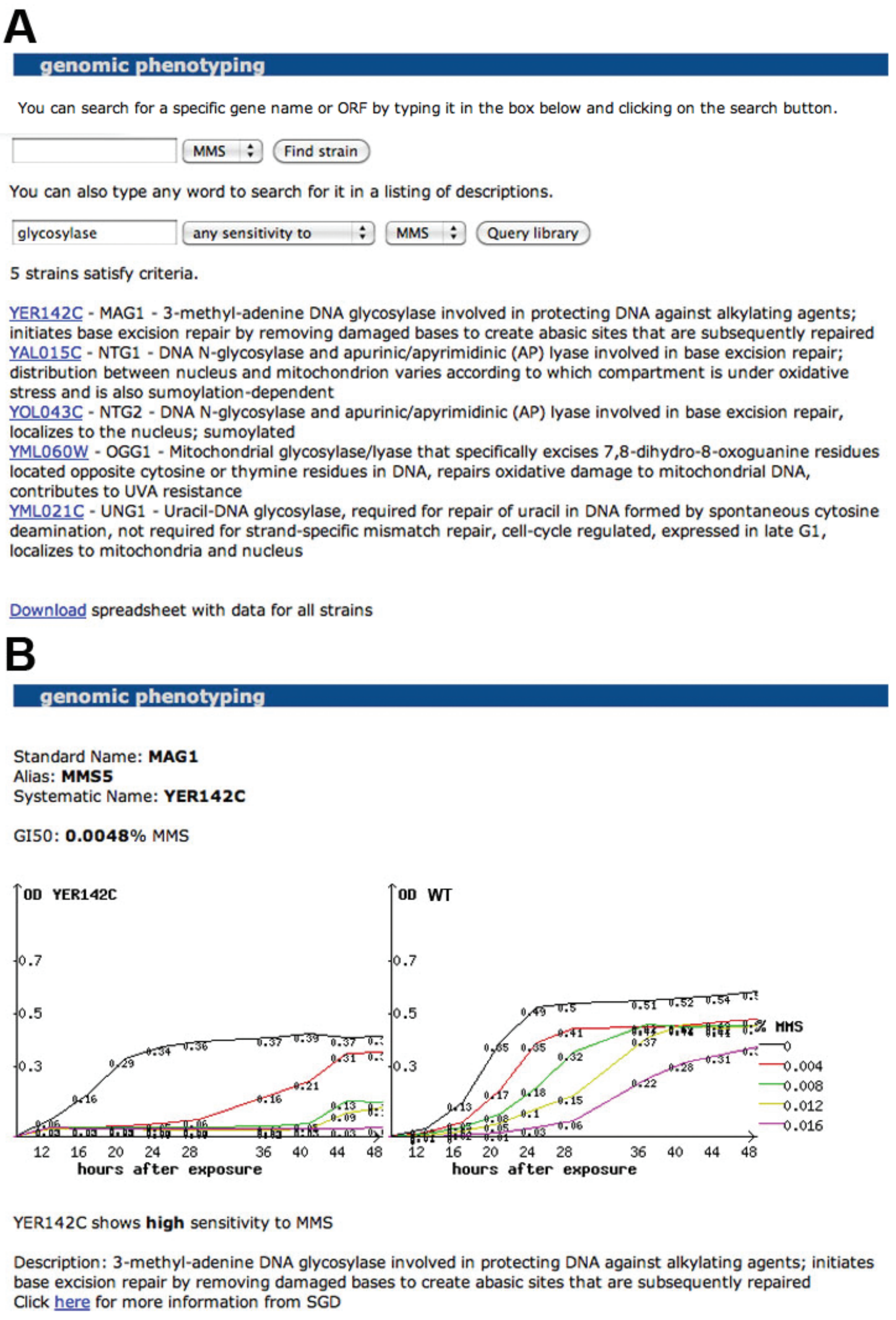

Figure S3. Screenshot of genomic phenotyping website. A) A searchable interface to find any queried strain by systematic name, common name or word used in SGD description (www.yeastgenome.org). B) The search results include both data from individual experiments and averaged data of queried strain and a comparison to the growth of the wildtype strain. Links to SGD and previous data on solid agar (Begley et al. 2004) are provided for additional information.

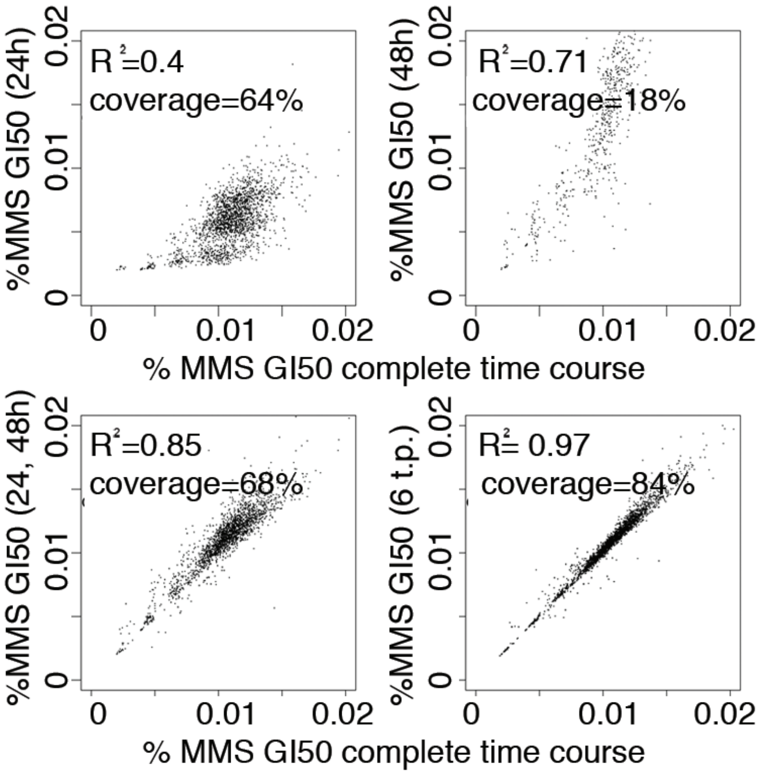

Figure S4. Scatterplots showing the influence of the sensitivity measurements when data composed of a selection of time-points are compared to the full time-course experiment. The tested series are 24h only, 48h only, 24 and 48h and 6 time points (t.p.) i.e. 16, 20, 24, 40, 44, 48h. The goodness-of-fit (R^2) between the two data sets and the coverage as calculated by the percentage of strains passing the linearity criterion (R^2>0.7 in at least two replicates) for the dose-response.
